# Non-invasive Drug Monitoring of β-Lactam Antibiotics Using Sweat Analysis—A Pilot Study

**DOI:** 10.3389/fmed.2020.00476

**Published:** 2020-08-25

**Authors:** Noé Brasier, Andreas Widmer, Michael Osthoff, Markus Mutke, Fiorangelo De Ieso, Pascale Brasier-Lutz, Lisa Wolfe, Vikas Aithal, Corey D. Broeckling, Jessica Prenni, Jens Eckstein

**Affiliations:** ^1^CMIO Research Group, University Hospital Basel, Basel, Switzerland; ^2^Department of Internal Medicine, Kantonsspital Obwalden, Sarnen, Switzerland; ^3^Department of Infectious Disease and Hospital Epidemiology, University Hospital Basel, Basel, Switzerland; ^4^Department of Internal Medicine, University Hospital Basel, Basel, Switzerland; ^5^Department of Gynaecology, Standort Wolhusen Kantonsspital Luzern, Wolhusen, Switzerland; ^6^Proteomics and Metabolomics Facility, Colorado State University, Fort Collins, CO, United States; ^7^Department of Horticulture and Landscape, Colorado State University, Fort Collins, CO, United States

**Keywords:** sweat, therapeutic drug monitoring, antimicrobial resistance, antibiotics, cefepime, imipenem, flucloxacillin

## Abstract

**Background:** Antimicrobial resistance is a major challenge in treating infectious diseases. Therapeutic drug monitoring (TDM) can optimize and personalize antibiotic treatment. Previously, antibiotic concentrations in tissues were extrapolated from skin blister studies, but sweat analyses for TDM have not been conducted.

**Objective:** To investigate the potential of sweat analysis as a non-invasive, rapid, and potential bedside TDM method.

**Methods:** We analyzed sweat and blood samples from 13 in-house patients treated with intravenous cefepime, imipenem, or flucloxacillin. For cefepime treatment, full pharmacokinetic sampling was performed (five subsequent sweat samples every 2 h) using ultra-high-performance liquid chromatography coupled with triple quadrupole mass spectrometry. The ClinicalTrials.gov registration number is NCT03678142.

**Results:** In this study, we demonstrated for the first time that flucloxacillin, imipenem, and cefepime are detectable in sweat. Antibiotic concentration changes over time demonstrated comparable (age-adjusted) dynamics in the blood and sweat of patients treated with cefepime. Patients treated with standard flucloxacillin dosage showed the highest mean antibiotic concentration in sweat.

**Conclusions:** Our results provide a proof-of-concept that sweat analysis could potentially serve as a non-invasive, rapid, and reliable method to measure antibiotic concentration and as a surrogate marker for tissue penetration. If combined with smart biosensors, sweat analysis may potentially serve as the first lab-independent, non-invasive antibiotic TDM method.

## Introduction

Antimicrobial resistance (AMR) is significantly impacting the prevention and treatment of infectious diseases on a global scale (1). The misuse and overuse of antibiotics (ABs) accelerate the development of AMR (2). Multidrug-resistant organisms (MDROs) threaten global health, food security, and development (2). Data from the European Antimicrobial Resistance Surveillance Network (EARS-Net) suggests that MDROs were responsible for approximately >670,000 infections and >30,000 deaths in the European Union in 2015 (3). Infections caused by AB-resistant bacteria are associated with a higher rate of complications and require significantly more resources than those from non-resistant microbes (2). Lee et al. (4) estimated the cost difference to be $7,070–$20,489 per case between community-associated methicillin-resistant *Staphylococcus aureus* and methicillin-susceptible *S. aureus*. Solutions to tackle AMR include antimicrobial stewardship measures, such as optimizing AB dosing.

Because of the associated antimicrobial toxicity, therapeutic drug monitoring (TDM) is standard for the treatment with glycopeptides and aminoglycosides. Studies have demonstrated the valuable effects of TDM on clinical outcomes, resulting in faster microbial eradication and lesser nephrotoxicity (5, 6). ABs with a wider therapeutic index than those of relatively toxic agents have not been thoroughly studied, despite their harmful propensity to accumulate in the case of kidney injury (7). Therefore, TDM is recommended in patients treated with cefepime who have kidney injury and a bacterial minimal inhibitory concentration (MIC) >8 mg/L (8). Critical illness, multi-morbidity, age, and polypharmacy further affect AB pharmacokinetics and pharmacodynamics (7, 9–12). Therefore, appropriate dosing becomes crucial but remains an unsolved issue (11). Recent efforts to develop a fast TDM technique using mass spectrometry for serum sample analysis have shown promising results (13, 14) but remains invasive, expensive and lab-independent. However, although TDM may improve the therapeutic target attainment, an easy-to-use, reliable, non-invasive, and laboratory-independent TDM is still lacking (15, 16).

Sweat is one of the most under-analyzed biological fluids, despite its content of different proteins, hormones, and metabolites (17, 18). Høiby et al. detected ciprofloxacin and β-lactam ABs in the sweat of healthy study participants (19, 20). The molecular solubility (hydrophilic or lipophilic) as well as the molecular size have to be seen as two of the main physiological drivers of pharmacological secretion into sweat (21). Those drivers are of importance in relation to the different sweat collection methods such as the induced or passive sweat collection and the specific body collection area. Newly developed sensors, such as the on-skin spot-enzyme-linked immunosorbent assay (ELISA), detect digital biomarkers in sweat (22). These sensors facilitate laboratory-independent sweat analysis and may potentially shape the future of healthcare diagnostics.

Here we investigated the detectability of the β-lactam ABs cefepime, imipenem, and flucloxacillin in patients' sweat and to the best of our knowledge, sweat analysis was assessed as a potential non-invasive AB drug monitoring strategy for the first time.

## Materials and Methods

### Study Design and Ethics

We conducted an observational pilot study with inpatients recruited from the University Hospital Basel, Switzerland. The trial lasted from September 2018 to June 2019. The patient sample size was chosen to generate the first pilot data with a reasonable amount of resources and time frame. The Ethics Committee of Northwest and Central Switzerland (EKNZ Reg-ID 2018-01155) approved this study, and all patients provided written informed consent. The ClinicalTrials.gov registration number is NCT03678142.

### Inclusion and Exclusion Criteria

The study included patients who had been treated with intravenous β-lactam ABs (cefepime, imipenem, and flucloxacillin) for >24 h, had an estimated glomerular filtration rate (eGFR) >50 mL·min^−1^·1.73 m^(2)−1^ and were >18 years old. Patients were excluded if they were allergic to pilocarpine or were receiving continuous oxygen supplementation.

### Bio-Fluid Sampling

Sweat samples were collected 4 h after starting the last imipenem and flucloxacillin infusions. For cefepime, the sweat and blood samples were collected at baseline (BL, before) and 2, 4, 6, and 8 h after administration. Sweat was induced and collected using a standardized method. Briefly, local eccrine sweat glands of the volar lower arm were stimulated for 5 min using pilocarpine iontophoresis. Sweat samples were collected using a CE certified Macroduct™ sweat collector for ~30 min and transferred to storage containers on dry ice (23). Sweat samples were stabilized by adding 1 μL Halt™ protease inhibitor cocktail (100×) and stored at −80°C.

During sweat gland stimulation in patients administered cefepime, we simultaneously collected reference blood samples, which were transported on dry ice and stored at −80°C. Standard laboratory parameters were included in the analysis (creatinine, eGFR, ALT, AST, gamma-glutamyltransferase, alkaline phosphatase, and bilirubin), and demographic data, such as age, weight, medication, and vital signs, were collected. Bio-fluid samples were transported on dry ice to Colorado State University, CO, USA. Bio-fluid analysis was conducted by the Proteomic and Metabolomic Facility of Colorado State University, CO, USA.

### Quantification of ABs Using Ultra-High-Performance Liquid Chromatography Coupled With Triple Quadrupole Mass Spectrometry (UPLC-QqQ-MS)

The mass spectrometer was operated in the multiple reaction monitoring (MRM) mode. In this mode, a parent ion is selected by the first quadrupole, fragmented in the collision cell, and then a fragment ion(s) is selected by the third quadrupole. Product ions, collision energies, and cone voltages were optimized for each analyte by direct injection of individual synthetic standards and their stable isotope-labeled internal standards (IS). Samples were injected into a reverse phase UPLC column, and target analytes and IS samples were eluted at specific retention times. A calibration curve was generated using authentic standards of each compound, and their corresponding stable isotope-labeled IS in 50% methanol:50% artificial sweat (https://www.pickeringtestsolutions.com/AP-eccrine/). The peak area values of target analytes were normalized to those of the appropriate IS and were plotted against expected concentrations ranging from 0.28 to 1,500 ng/mL.

### AB Extraction From Human Whole Blood for LC-MS Analysis

To extract the ABs, blood samples were thawed on ice, and 100 μL of each was placed into a 2.0 mL polypropylene microfuge tube. Then, 900 μL ice-cold methanol (spiked with 275 ng/mL of cefepime-d3) was added to each sample and vortexed for 10 min at 4°C. Samples were stored at −80°C for a minimum of 1 h to facilitate protein precipitation. Precipitated proteins were collected by centrifugation at 15,000 × g for 20 min at 4°C, then 900 μL of the supernatant was collected into 2 mL autosampler vials.

### AB Extraction From Human Eccrine Sweat for LC-MS Analysis

Sweat samples (50 μL) were diluted 1:1 (v/v) with 100% methanol (spiked with 500 ng/mL meropenem-d6, cefepime d3, and flucloxacillin-13C4). Each sample was briefly vortexed, then 50 μL was transferred into autosampler vials.

### Standard Curve and Is Mixture

Authentic standards of imipenem, flucloxacillin, and cefepime were diluted from 1 mg/mL library stocks to a 10 μg/mL master mix. Isotopically-labeled IS samples were diluted from 1 mg/mL library stocks to a 2.5 μg/mL IS-Master Mix, which was further diluted with artificial sweat (Pickering Laboratories, Mountain View, CA, USA) at a concentration of 500 ng/mL (IS-Artificial Sweat). To construct the standard curve, the standard master mix in 100% methanol was serially diluted 3.2 times from 25 ng/mL to 0.28 ng/mL in 100% methanol. Then, 0.1 mL of each methanol dilution was added to 0.1 mL of IS-Artificial Sweat, and the final concentration of the IS was 250 ng/mL.

### UPLC-QqQ-MS Analysis

LC-MS/MS was performed using a Waters Acquity UPLC coupled with a Waters Xevo TQ-S QqQ mass spectrometer. Chromatographic separations were conducted using a Waters UPLC T3 C18 stationary phase (1 × 50 mm, 1.7 μM) column. Mobile phases were 99.9% acetonitrile with 0.1% formic acid (B) and 99.9% water with 0.1% formic acid (A). The analytical gradient schedule was as follows: 0 min, 0.1% B; 1.0 min, 0.1% B; 2.5 min, 99% B; 3.5 min, 99% B; 3.55 min, 0.1% B; and 5 min, 0.1% B. The flow rate was 800 μL/min and injection volume was 5 μL. Samples were held at 6°C in the autosampler, and the column was operated at 45°C. The MS was operated in positive ionization mode with the capillary voltage set to 3 kV. The inter-channel delay was set to 3 ms, and the source and desolvation temperatures were 150 and 500°C, respectively. Desolvation and cone gas flow rates were 1,000 and 150 L/h, while the collision gas flow was 0.2 mL/min. The nebulizer pressure (nitrogen) was set to 7 bar, and argon was the collision gas. The MS acquisition functions were scheduled by retention time and provided 30 s windows. Autodwell feature was set for each function, and the dwell time was automatically calculated, while 12 points across a peak were specified as the minimum data points per peak, resulting in a minimum dwell time of 0.017 s.

### Data and Statistical Analysis

All raw data files were imported into the Skyline open-source software package (24). Each target analyte was visually inspected for retention time and peak area integration. Peak areas were extracted for target compounds detected in biological samples and normalized to the peak area of the appropriate IS or surrogate in each sample. Normalized peak areas were exported to Excel, and absolute quantitation was obtained using the linear regression equation generated for each compound from the calibration curve. Limits of detection (LOD) and limits of quantification (LOQ) were calculated as 3 or 10 times the standard deviation (SD) of the blank divided by the slope of the calibration curve, respectively (25, 26).

### Quality Control (QC)

Study-specific quality control (QC) samples were generated to represent the pool of all samples. They were injected into the system after every seven samples were analyzed.

## Results

### Patients

**Figure 1:**
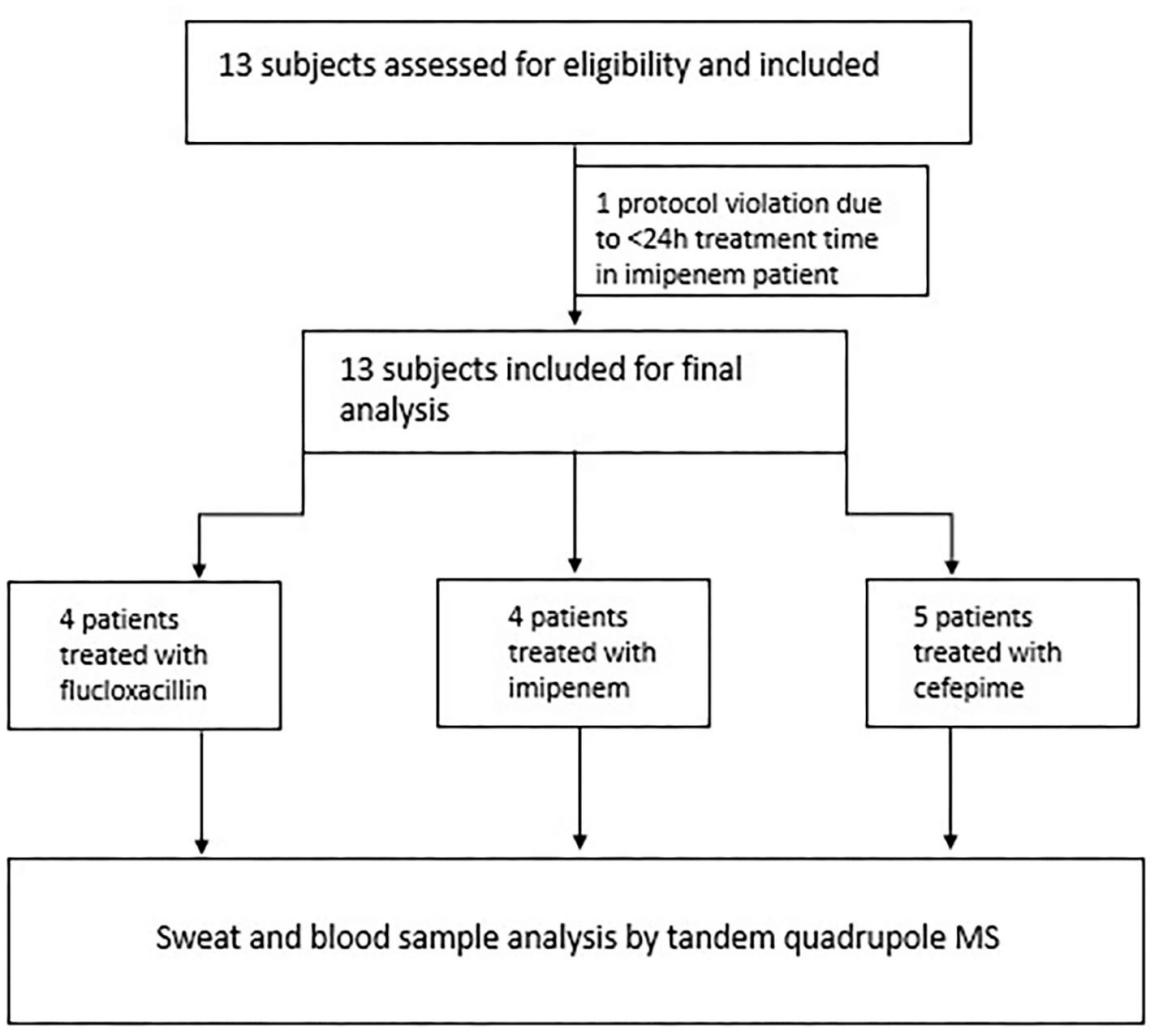
Patient flowchart: inclusion and exclusion of study patients.

We recruited 13 patients, from which four were treated with flucloxacillin and cefepime, and three were administered imipenem. One patient was treated with a combination of imipenem and cefepime and was included for both groups, but a protocol violation occurred for imipenem (AB treatment <24 h). However, the samples were still included because the study was observational (Figure 1). One cefepime sweat sample was lost because of sample mishandling; one pair of cefepime sweat and blood samples could not be collected because the patient did not attend the follow-up visit.

Patient characteristics are shown in Table 1 with a detailed summary available as Tables S1–S3. Patients were aged 34 to 90 years with kidney function (eGFR) values ranging from 58 to 124 mL·min^−1^·1.73 m^(2)−1^, and their indications for AB therapy varied from osteomyelitis to pneumonia.

**Table 1:** Study population characteristics.

**Characteristics**	**Flucloxacillin (*n* = 4)**	**Imipenem (*n* = 4)**	**Cefepime (*n* = 5)**	**Total (*n* = 13)**
Age (y), mean ± SD	63.5 ± 21.0	63.2 ± 19.5	65.6 ± 22.4	64.2 ± 19.4
Male sex, *n* (%)	2 (50)	2 (50)	3 (60)	7 (53.8)
GFR (ml/min/1.73 m^2^), mean ± SD	92.0 ± 15.25	96.5 ± 23.1	92.8 ± 25.4	93.7 ± 20.25
Body temperature (°C), mean ± SD	36.8 ± 0.5	36.9 ± 0.7	37.1 ± 0.4	36.8 ± 0.5
Chronic kidney disease, *n* (%)	0 (0)	1 (25)	1 (20)	2 (15.4)
Diabetes mellitus, *n* (%)	0 (0)	2 (50)	1 (20)	3 (23.1)
Number of comorbidities	4	11	7	22
CRP (mg/l), mean ± SD	114.9 ± 99.0	135.65 ± 123.93	74.9 ± 88.4	105.9 ± 98.06

*y, years; n, number; GFR, glomerular filtration rate; CRP, c-reactive-protein; SD, standard deviation*.

### Detection of ABs in Sweat

In this study, cefepime, flucloxacillin, and imipenem were detected for the first time in all of the respective human eccrine sweat (Tables 2–4). Sweat sample volumes ranged from 25 to 50 μl.

1. Patients treated with cefepime had a mean AB concentration of 0.31 and 32.72 mg/L in sweat and blood, respectively. Cefepime was detected in all sweat samples of patients receiving AB (Table 2).
2. Patients treated with flucloxacillin showed a mean AB concentration of 0.83 mg/L 4 h in sweat after the start of the infusion. Flucloxacillin was detected in all sweat samples of patients receiving AB (Table 3).
3. Patients treated with imipenem exhibited a mean AB concentration of 0.04 mg/L 4 h in sweat after the start of the infusion. Imipenem was detected in all sweat samples of patients receiving AB (Table 4).

**Table 2:** Antibiotic concentrations in the sweat and blood of patients treated with cefepime.

**Cefepime concentration (mg/L)**	**Patient 1 (C1)**	**Patient 2 (C2)**	**Patient 3 (C3)**	**Patient 4 (C4)**	**Patient 5 (C5)**
**SWEAT**					
**Time**					
BL	1.253	0.080	0.011	0.036	0.368
BL+2 h	3.422	0.041	0.045	NA	0.768
BL+4 h	0.215	0.029	0.033	0.096	0.094
BL+6 h	0.074	0.007	0.016	NA	0.133
BL+8 h	0.785	0.023	0.022	0.060	0.099
Mean	1.150	0.036	0.025	0.065	0.292
**BLOOD**					
**Time**					
BL	20.285	3.947	7.613	5.824	159.948
BL+2 h	67.016	33.630	18.599	34.667	59.736
BL+4 h	42.930	20.713	23.421	10.142	59.929
BL+6 h	38.587	12.321	14.967	NA	63.684
BL+8 h	37.233	13.157	13.728	3.958	38.415
Mean	41.210	16.754	15.666	13.648	76.342

*BL, baseline before antibiotic treatment application; NA, not available*.

**Table 3:** Antibiotic concentrations in the sweat of patients treated with flucloxacillin.

**Flucloxacillin concentration (mg/L) in sweat**	**Patient 1 (F1)**	**Patient 2 (F2)**	**Patient 3 (F3)**	**Patient 4 (F4)**	**Mean**
**TIME**					
BL+4 h	0.059	0.082	0.877	2.290	0.827

*BL, baseline before antibiotic treatment initiation*.

**Table 4:** Antibiotic concentrations in the sweat of patients treated with imipenem.

**Imipenem Concentration (mg/L) in sweat**	**Patient 1 (I1)**	**Patient 2 (I2)**	**Patient 3 (I3)**	**Patient 4 (I4)**	**Mean**
**TIME**					
BL	NA	NA	NA	0.004	0.004
BL+4 h	0.014	0.037	0.050	0.052	0.038
BL+8 h	NA	NA	NA	0.046	0.046

*BL, baseline before antibiotic treatment initiation; NA, not available*.

The highest interpersonal AB sweat concentration variation was detected between patients treated with cefepime at 2 h with values of 0.041 mg/L (Patient C2) compared to 3.422 mg/L (Patient C1), corresponding to a variation factor of 84 (Table 2). The lowest interpersonal AB concentration variation was found between patients treated with imipenem with a variation factor of 3.6 (Table 4).

### Pharmacokinetic Monitoring of Cefepime in Sweat

**Figure 2:**
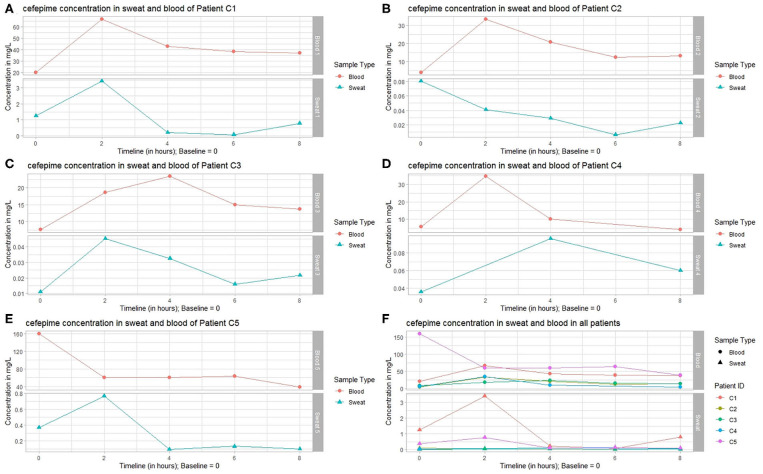
Detection of cefepime: sweat and blood concentrations of patients treated with cefepime over time. Sweat and blood concentrations of **(A)** patient 1, **(B)** patient 2, **(C)** patient 3, **(D)** patient 4, and **(E)** patient 5; **(F)** Overview of the sweat and blood concentrations of patients 1–5.

Cefepime AB concentration changes over time showed similar dynamics in blood and sweat (Figures 2A–F). The mean AB concentration of cefepime in sweat and blood ranged from 0.036 to 1.15 mg/L and 13.648 to 76·342 mg/L, respectively (Table 2). Sweat concentrations showed the highest variation 2 h after BL with a factor of 84 (Patients C1 and C2, Table 2) with comparable dynamics in concentration changes (Figure 2A). Patient C2 showed similar trends in samples analyzed at 2, 4, 6, and 8 h after BL (Figure 2B). In patient C3, after an earlier increase in sweat cefepime concentrations compared to blood concentrations, samples analyzed 4, 6, and 8 h after BL showed simultaneous changes in both sweat and blood. Importantly, patient C3 was administered the lowest cumulative daily AB dose (single dose of 1,000 mg at BL), which resulted in the lowest mean AB concentration in sweat and a prolonged concentration peak in the blood (Figure 2C). Patient C5 showed the highest total mean concentration of cefepime in the blood (76.342 mg/L), but concentration changes in blood and sweat of patient C5 showed a delay of 2 h compared to those of patients C1, C2, and C3 (Figure 2E).

In all patients treated with cefepime, the interpersonal factor of variability derived from the maximum peak concentration variability was higher in sweat (3.422 [maximum]/0.041 mg/L [minimum]) = 84 [factor]) than in blood (159.948/3.947 mg/L = 41). Analysis of the highest variation of trough levels detected in patients showed a slightly higher interpersonal concentration variability with sweat (0.074/0.007 mg/L = 11) than with blood (38.415/3.947 mg/L = 10). By dividing the patients into two groups based on age (>60 years) and kidney function (eGFR < 85 mL·min^−1^·1.75 m^(2)−1^), the concentration variability factor reduced substantially for maximum and trough levels. The maximum concentration variability factor of sweat decreased from an initial value of 84 to 2 (0.096/0.045 mg/L) in the younger patients and 5 (3.422/0.768 mg/L) in older patients. In blood samples, the maximum concentration variability factor decreased from 40 to 1 (34.667/33.630 mg/L) in young patients and 2 (159.948/67.016 mg/L) in older patients. A comparison of patient trough levels between the two age groups showed a sweat concentration variability factor of 2 (0.011/0.007 mg/L) in young patients and 1 (0.094/0.074 mg/L) in older patients. Serum trough levels showed a cefepime concentration variability factor of 1 (3.958/3.947 mg/L) in younger patients and 2 (38.415/20.285 mg/L) in older patients. Further, the gap between mean blood and sweat AB concentrations was substantially smaller in the older patients (patient C1: 41.210/1.150 mg/L = 36, patient C5: 76.342/0.292 mg/L = 261) than in younger patients (patient C2: 16.754/0.036 mg/L = 465, patient C3: 15.666/0.025 mg/L = Factor 627).

The elimination rate constant (K_e_) showed comparable values in sweat and blood in younger patients with adequate GFR (patient 2: K_e_ was 0.16/h in sweat and 0.17/h in blood, patient 3: K_e_ was 0.14/h in sweat and 0.13/h in blood, patient 4: K_e_ was not calculated due to missing values). In older patients, K_e_ diverged widely compared to younger patients, and showed the widest divergence in the oldest patient (patient 1: K_e_ was 0.27/h in sweat and 0.09/h in blood, patient 5: K_e_ is 0.22/h in sweat and 0.14/h in blood).

## Discussion

This pilot study evaluated the potential of a novel non-invasive AB drug monitoring strategy by sweat analysis in patients treated with intravenous β-lactam ABs. We demonstrated that cefepime, flucloxacillin, and imipenem were detectable in 100% of patient sweat samples.

Flucloxacillin showed the highest mean AB concentration in sweat. This was expectable because the main indications for flucloxacillin are bacterial soft tissue infections due to its established soft tissue penetration (27). Our novel approach to AB treatment monitoring of soft tissue infections could enable personalized treatment dosing. It could also ensure adequate local AB concentrations above the minimal inhibitory concentration (MIC) of the pathogen, which may improve patient outcomes. The European Committee on Antimicrobial Susceptibility Testing (EUCAST) has increasingly investigated and proposed clinical MIC breakpoints of bacterial strains, which may be used as target concentrations for TDM by sweat analysis (28).

Interpersonal concentration variability of cefepime in blood and sweat is considered very low when considering age and GFR. It is important to point out that all cefepime concentrations in blood samples of the older patient group were >20 mg/L, including trough plasma levels, which is associated with severe toxicities (29). This further indicates the need for personalized AB TDM not only to prevent the development of resistance but also to protect patients from toxic side effects. The varying mean cefepime concentrations in the sweat and blood of patients of different ages may be partially explained by lower main body water volume and different sweat patterns between different age groups, suggesting that patient age and GFR should be taken into consideration for dose optimization (30). The wider divergence of K_E_ in sweat and blood along older patients with lower GFR needs to be further considered, as AB concentrations in sweat may underestimate blood concentrations in older patients. This will need further evaluation and testing. The only patient co-treated with cefepime and imipenem showed imipenem concentrations in sweat after the first treatment and, therefore, potentially qualifies as an AB monitoring that can begin at treatment initiation.

Smartphone-based sweat sensor technologies are under development and will soon be available for clinical testing and implementation (31). Once they are available, this non-invasive AB monitoring tool would be a highly promising resource for on-demand personalized AB treatment. In combination with smartphones, these biosensors would enable laboratory-independent drug monitoring in sweat, which can be used without the need for direct health care access (31).

Despite the promising results, this study has a few limitations. Pilocarpine iontophoresis was conducted under standardized conditions for 5 min, followed by sweat sampling for 30 min. Differences occur in sweat volume and composition of electrolytes if excessive sweating is prolonged. Therefore, a more advanced method that would enable spot measurements is needed to verify these results. Combining the analysis of sweat with that of vital signs such as body temperature and sweat rate would allow a more standardized sampling approach.

Despite instant sample cooling, the on-skin sweat sampling procedure and the analysis processing times should be considered during the interpretation of results. Cefepime is known to be unstable at body temperature (32); therefore, absolute sweat concentrations should be carefully interpreted and confirmed using spot measurements. Nevertheless, the detection of concentration changes of cefepime in sweat and blood samples remains highly promising.

The Macroduct sweat collector is an established medical device that actively stimulates sweat glands using pilocarpine iontophoresis with a standardized protocol (23). Although this is the most standardized sampling modality, especially in patients, it is not clear whether activating sweat glands is the most reliable procedure. It is of importance in regard to different medication characteristics such as the lipid solubility. As actively induced eccrine sweat glands secret basically water- and salt-based liquids, an actively induced sweat gland stimulation may not be the best approach for lipophilic compounds (33). Further investigations of active and passive sweat sampling is needed.

Due to internal sample processes, blood samples were directly frozen after sampling, and vortexed along thawing processes during sample analysis. β-lactam antibiotics are very unlikely to accumulate intracellularly (34). Therefore, we assume that human intracellular concentrations are negligible for this first pilot project.

Finally, the sample size was very small, and more patients need to be investigated. Nevertheless, this novel approach has high potential to serve as the first non-invasive AB monitoring tool. Trials correlating AB sweat concentrations with clinical outcome to verify the efficacy of sweat analysis in AB TDM are needed.

This study revealed the high potential of sweat analysis to revolutionize antimicrobial TDM. AB concentration changes in sweat and blood showed comparable dynamics and were quickly detectable. Considering the heterogeneity of the patient population, sweat analysis—in combination with next-generation smartphone-based biosensors—is a novel and highly promising approach. Further, the detection of ABs in sweat could serve as an indicator of local tissue concentrations that could significantly impact the prevention and treatment of soft tissue infections.

## Data Availability Statement

The raw data supporting the conclusions of this article will be made available by the authors, without undue reservation, to any qualified researcher.

## Ethics Statement

The Ethics Committee of Northwest and Central Switzerland (EKNZ Reg-ID 98 2018-01155) approved this study, and all patients provided written informed consent.

## Author Contributions

NB, JE, AW, JP, LW, and PB-L conceived and designed the study. NB and JE coordinated and performed the study. NB, JE, MO, MM, FD, AW, JP, CB, LW, and VA collected and analyzed the data. NB, FD, JE, MO, AW, and CB wrote the manuscript. All authors made a substantial contribution to the manuscript and approved the final manuscript version.

## Conflict of Interest

MO received a project grant and consulting fees from Pharming Biotechnologies B.V. with regards to a different project. The remaining authors declare that the research was conducted in the absence of any commercial or financial relationships that could be construed as a potential conflict of interest.
