# Supplementary material for: Non-invasive Drug Monitoring of β-Lactam Antibiotics Using Sweat Analysis—A Pilot Study
Source: Front Med (Lausanne). 2020 Aug 25;7:476. doi: 10.3389/fmed.2020.00476 (PMC7477313; doi:10.3389/fmed.2020.00476)
Supplement: Supplementary file 1 [file Table_1.DOCX]

| Patient | C1 | C2 | C3 | C4 |  | C5 |  |
| --- | --- | --- | --- | --- | --- | --- | --- |
| Age (years) | 90 | 52 | 73 | 34 |  | 79 |  |
| Gender (male/female) | m | m | f | m |  | f |  |
| Weight (kg) | 69.0 | 73.3 | 119.0 | 102.0 |  | 62.0 |  |
| Body temperature (°C) | 36.9 | 37.5 | 36.8 | 36.8 |  | 37.6 |  |
| Creatinine (µmol/l) | 98 | 59 | 57 | 60 |  | 57 |  |
| GFR (ml/min/1.73m^2) | 58 | 110 | 88 | 124 |  | 84 |  |
| Leukocyte count (x10^9/L) | 8.37 | 3.35 | 5.90 | 10.13 |  | 7.73 |  |
| C-reactive protein (mmg/L) | 60.0 | 2.6 | 227.5 | 29.1 |  | 55.1 |  |
| ALAT (U/l) | 24 | 27 | 31 | 48 |  | 37 |  |
| ASAT (U/l) | 38 | 42 | 23 | 42 |  | 28 |  |
| AP (U/l) | 95 | 78 | 154 | 199 |  | 131 |  |
| Bilirubin (µmol/l) | 6.1 | 5.1 | 2.4 | 9.4 |  | 3.0 |  |
| yGT (U/l) | 32 | 45 | 262 | 212 |  | 145 |  |
| Infusion (yes/no) | no | no | yes | yes |  | no |  |
| Infusion rate (ml/h) | NA | NA | 21 | 10 |  | NA |  |
| Infusion content | NA | NA | Ringer’s lactate | 0.9%  Sodium  chloride |  | NA |  |
| Cefepime: |  |  |  |  |  |  |  |
| - Application per dose (mg) | 2000 | 2000 | 1000 | 2000 |  | 2000 |  |
| - per day (mg) | 4000 | 4000 | 3000 | 4000 |  | 4000 |  |
| - amount of previous doses (n) | 5 | 5 | 5 | 13 |  | 28 |  |
| Amount of comorbidities | 0 | 1 | 5 | 0 |  | 1 |  |
| Medication: |  |  |  |  |  |  |  |
| Acetaminophen (yes/no) | yes | no | no | no |  | no |  |
| Metamizol (yes/no) | no | no | no | no |  | no |  |
| Beta blocker (yes/no) | no | yes | no | no |  | no |  |
| Diuretics (Yes/No) | yes | no | no | no |  | no |  |

**Table S1:** Population characteristics of patients treated with cefepime; NA indicates not available

| Patient | F1 | F2 | F3 | F4 |
| --- | --- | --- | --- | --- |
| Age (years) | 76 | 41 | 51 | 86 |
| Gender (male/female) | m | f | m | m |
| Weight (kg) | 108.0 | 73.0 | 89.1 | 66.2 |
| Body temperature (°C) | 36.5 | 37.5 | 36.4 | 36.8 |
| Creatinine (µmol/l) | 57 | 66 | 70 | 82 |
| GFR (ml/min/1.73m^2) | 86 | 99 | 109 | 74 |
| Leukocyte count (x10^9/L) | 13.35 | 2.58 | 5.67 | 10.43 |
| C-reactive protein (mg/L) | 219.5 | 59.5 | 5.8 | 174.4 |
| ALAT (U/l) | 15 | 36 | 77 | 38 |
| ASAT (U/l) | - | 22 | 30 | 58 |
| AP (U/l) | 54 | 104 | 80 | 104 |
| Bilirubin (µmol/l) | 5.2 | 3.2 | 5.0 | 6.9 |
| yGT (U/l) | 50 | 104 | 96 | 43 |
| Infusion (yes/no) | yes | no | no | no |
| Infusion rate (ml/h) | 21 | NA | NA | NA |
| Infusion content | NaCl | NA | NA | NA |
| Flucloxacillin: |  |  |  |  |
| - Application per dose (mg) | 2000 | 2000 | 2000 | 2000 |
| - per day (mg) | 12000 | 12000 | 12000 | 10000 |
| - amount of previous doses (n) | 25 | 15 | 22 | 36 |
| Amount of comorbidities | 1 | 1 | 0 | 2 |
| Medication: |  |  |  |  |
| Acetaminophen (yes/no) | no | no | no | no |
| Metamizole (yes/no) | no | no | no | no |
| Beta blocker (yes/no) | yes | no | no | yes |
| Diuretics (yes/no) | yes | no | no | no |

**Table S2:** Population characteristics of patients treated with flucloxacillin

| Patient | I1 | I2 | I3 | I4 |
| --- | --- | --- | --- | --- |
| Age (years) | 73 | 73 | 73 | 34 |
| Gender (male/female) | m | f | f | m |
| Weight (kg) | 63.3 | 88.7 | 119.0 | 102.0 |
| Body temperature (°C) | 36.6 | 37.7 | 36.4 | 36.8 |
| Creatinine (µmol/l) | 49 | 74 | 53 | 60 |
| GFR (ml/min/1.73m^2) | 103 | 69 | 90 | 124 |
| Leukocyte count (x10^9/L) | 12.45 | 8.51 | 8.10 | 10.13 |
| C-reactive protein (mg/L) | 298.0 | 166.1 | 49.4 | 29.1 |
| ALAT (U/l) | 10 | 24 | 32 | 48 |
| ASAT (U/l) | 8 | - | 35 | 42 |
| AP (U/l) | 80 | 67 | 209 | 199 |
| Bilirubin (µmol/l) | 8.1 | 8.0 | 2.4 | 9.4 |
| yGT (U/l) | 137 | 37 | 244 | 212 |
| Infusion (yes/no) | yes | no | yes | yes |
| Infusion rate (ml/h) | 10 | NA | 10 | 10 |
| Infusion content | Ringer | NA | Glucose 5% | NaCl |
| Imipenem: |  |  |  |  |
| - Application per dose (mg) | 500 | 500 | 500 | 500 |
| - per day (mg) | 2000 | 2000 | 1500 | 2000 |
| - amount of previous doses (n) | 26 | 4 | 6 | 0 |
| Amount of comorbidities | 3 | 3 | 5 | 0 |
| Medication: |  |  |  |  |
| Acetaminophen (yes/no) | no | no | no | no |
| Metamizole (yes/no) | no | no | yes | yes |
| Beta blocker (yes/no) | yes | no | no | no |
| Diuretics (yes/no) | no | no | no | no |

**Table S3:** Population characteristics of patients treated with imipenem
